# Detection of effective factors of accidents based on metal patters of urban drivers using Q-analysis

**Published:** 2019-08

**Authors:** Ali Nasrollah Tabar, Mahmoud Reza Keymanesh, Elnaz Arghand, Behzad Mohammadi

**Affiliations:** ^*a*^Arian Science and Technology Institute, Tehran, Iran.; ^*b*^Faculty member of Payam Noor University of Tehran, Tehran, Iran.; ^*c*^Payam Noor University of Tehran, Tehran, Iran.

## Abstract

**Background:**

The detection of effective factors of accidents is a major step in increasing the level of road safety and reducing the casualties, particularly in moderate-income countries. The research set conducted about the effective factors of accidents in Iran has focused on the view of the police or road experts and mental patterns of drivers have not been considered as one of the main users in regard to the cause of accidents. Aimed at the detection of attitude of urban drivers, this study is carried out based on mental patterns of taxi drivers in Tehran using Q-factor analysis.

**Methods:**

Given the previous studies and research discourse space, 54 propositions are extracted after summarization and the Q-factor analysis is done by selecting 30 urban drivers through purposive sampling and collecting their opinions, where 7 mental categories are derived.

**Results:**

The opinions of urban drivers suggest that human factors, e.g. overtaking, deviation to the left, talking on cellphone, driver’s problems in spite of his/her driving skill and speeding, have the maximum impact on urban accidents variance ratio criterion (VRC), while lack of road monitoring by the police and illegal passenger pickup have the minimum effect on the cause of car crashes.

**Conclusions:**

It is concluded that the drivers’ image of effective factors of accidents is not the same as the results of safety monitoring. The detection of these patterns helps the experts to modify their opinions and it is possible to correct misguided mental patterns of drivers about the cause of accidents by encouraging the educational processes.

**Keywords:**

Accidents, Urban drivers, Q methodology

